# Health and Productivity Management in Hospital Organizations and Work Engagement of Nurses

**DOI:** 10.14789/jmj.JMJ23-0020-OA

**Published:** 2023-12-22

**Authors:** YUMI ARAI, KENTARO INABA, TAKUMI IWAASA, YASUYUKI HOCHI, YUKI MIZUNO, MOTOKI MIZUNO

**Affiliations:** 1 Graduate School of Health and Sports Science, Juntendo University, Chiba, Japan; 1 Graduate School of Health and Sports Science, Juntendo University, Chiba, Japan; 2 Ishinomaki Senshu University, Miyagi, Japan; 2 Ishinomaki Senshu University, Miyagi, Japan; 3 Japan Women's College of Physical Education, Tokyo, Japan; 3 Japan Women's College of Physical Education, Tokyo, Japan

**Keywords:** health, productivity management, hospital organization, nurses, work engagement

## Abstract

**Objective:**

In Japan, there is an urgent need to strengthen efforts to retain nurses and prevent high turnover. The Japan Nurses Association has set the goal of creating a supportive work environment for nurses to work with peace of mind and improve outcomes throughout their lives. Against this background, we examined the relationship between nurses’ health and productivity management and their work engagement (WE) in Japanese hospital organizations.

**Design:**

A cross-sectional design was used.

**Methods:**

A web-based survey was administered to full-time employed nurses working in Japanese hospitals with 100 or more beds.

**Results:**

Total WE scores were analyzed as the objective variable; WE crude odds ratios (ORs) were significantly higher in the high group than in the low group for all indicator items related to a healthy workplace culture. For adjusted ORs, propensity scores were calculated from gender, age, years of service, years of experience, job title, marital status, work shift, frequency of exercise per week, and hours worked per week and used as moderator variables. The results showed that the adjusted ORs for the high group were significantly higher than the adjusted ORs for the low group for all items except “participation of the person in charge from the planning stage of the initiative” and “reflection of the person in charge’s opinion in the planning of the initiative.”

**Conclusions:**

This study suggests that health and productivity management initiatives in hospital organizations may positively impact nurses' WE. Furthermore, it suggests that these initiatives may contribute to improving nurse retention and preventing turnover.

## Introduction

Nurses (excluding assistant nurses) working in hospitals, clinics, and as care workers exceed 1.33 million in Japan^1)^. Since 2008, Japan's average nursing attrition rate has remained at 10-11%^2)^; therefore, there is an urgent need to strengthen efforts to retain and prevent high turnover among Japanese nurses. The Japanese Nursing Association has developed an occupational safety and health guideline^3)^ to ensure healthy and safe workplaces, as well as promote environments that enable nursing staff to engage in health promotion. In 2021, as a work style reform, the Japanese government set the goal of creating work environments that support nurses in working at ease and improve outcomes throughout their working lives^4)^. In this new policy effort, the turnover problem is approached from the perspective of health promotion, and the aim is to maintain nurses' morale so that they continue to work in their current position. It also aims to ensure that nurses' workplaces support them in maintaining their physical and mental well-being.

The concept of health and productivity management, proposed by Rosen^5)^, has been attracting considerable attention. This is because, from a managerial perspective, placing focus on employee health management is expected to have multiple positive outcomes^6)^.

Previous studies identifying the relationship between employee health status and productivity have shown that health risks are increasingly associated with lower productivity^7, 8)^. A particularly notable finding was that, “Physical activity may have a direct and indirect relationship to quality resident care and long-term care organizational success through impacts on employee absenteeism, job performance and employee satisfaction”^9-12)^. Another study found that, “Physical activity level may also indirectly impact resident care through employee job satisfaction. A small increase in physical activity has been shown to increase healthcare employee job satisfaction”^10)^. Given these trends, medical organizations and social medical corporations are making efforts to promote health and productivity management^13)^.

The concept of work engagement (WE) has been defined as individuals' “positive, fulfilling, work- related state of mind that is characterized by vigor, dedication, and absorption”^14)^. Thus, WE can be used as an index of an individual's positive and energized state of mind toward work. It is characterized by “vigor, dedication, and absorption”^14)^ toward work and higher WE reportedly has positive outcomes, such as increased happiness, job satisfaction, and organizational commitment^15, 16)^. Higher WE among nurses reduces their risk of impaired physical and mental health, enabling them to contribute more to their work^17-19)^. One prior study also emphasized the importance of further analyzing the current state of nurses' WE and its driving factors^20)^.

Despite the emphasis on the Japanese context above, concerns related to the shortage and turnover of nurses are not unique to Japan^21)^. For example, the American Nurses Association^22)^ works to prevent high turnover by advocating safe and ethical work environments, staffing levels, and the physical and emotional well-being of nurses. The Healthy Nurse, Healthy Nation^TM^^23)^ program of the American Nurses Association Enterprise encompasses six domains (mental health, physical activity, nutrition, rest, quality of life, and safety) and serves as an action plan to improve nurses' health. These efforts related to health and productivity management are similar to those in Japan. In the latter case, the certification system for health and productivity management is a mechanism for evaluating systems, programs, and management that promote health maintenance at the corporate level, and this system evaluates some aspects of corporate health climate and culture^24)^. WE has been suggested to be associated with factors of corporate health culture. Furthermore, WE research has demonstrated the positive correlation among support from supervisors and colleagues, coaching by supervisors, innovative environment, and congruence between organizational and individual values^25)^.

Furthermore, previous studies suggest a close association between Japanese nurses' exercise habits and job satisfaction/intention to continue working^26)^. Therefore, efforts made by hospital organizations to improve health and productivity management by encouraging health activities such as exercise and sports are expected to improve nurses' WE and retention and prevent turnover. We examine the relationship between nurses' perceptions of health and productivity management in hospital organizations and their WE. Particular attention will be paid to identifying health culture that contributes to nurses having high WE.

## Materials and Methods

### Procedure and sample

#### Sampling

A web-based survey was conducted among full-time employed nurses, midwives, and public health nurses working in Japanese hospitals with more than 100 beds. The sample comprised 500 respondents selected from the panel registered with Cross Marketing, Inc., a leading marketing research agency in Japan. The survey period was from March 1 to April 18, 2022, and 518 responses were initially included in the study. After excluding those with extremely short response times (under four minutes) and samples with the same response to a single question (straight line cut), 377 answers were selected for the final analysis (valid response rate: 72.8%).

#### Attributes of participants

The participants' attributes are shown in Table 1. There were 65 men (17.2%) and 312 women (82.8%). The mean age was 43.1 (± 9.6) years, consistent with the volume zone of nurses' age, 40-44 years (14.2%), as reported by the Ministry of Health, Labor and Welfare^1)^. The number of years of nursing experience was 18.6 (± 9.5), and the number of years of service in the current position was 12.6 (± 8.7). Furthermore, 268 (71.1%) participants were in the staff class, 81 (21.5%) were in the supervisor class, 16 (4.2%) were in the head nurse class, and 12 (3.2%) were in the director class. The percentage of positions was also consistent with the Japanese Nursing Association survey^4)^, with 73.0% of the respondents in non-managerial positions (staff class) and 22.8% in middle-managerial positions (chiefs and division chiefs).

**Table 1:** Participants' attribute scores

	n	%	Mean	SD
Gender				
Men	65	17.2		
Women	312	82.8		
Age			43.1	8.7
Years of nursing experience			18.6	9.5
Years of service at the current workplace			12.6	8.7
Position				
Staff class	268	71.1		
Chief class	81	21.5		
Manager class	16	4.2		
Director class	12	3.2		
Marital status				
Unmarried	120	31.8		
Married (including separated and widowed)	257	68.7		
Work shifts				
Day shift only	88	23.3		
Two-shift system	216	57.3		
Three-shift system	68	18.0		
Night shift only	5	1.3		
Frequency of exercise				
Less than 1 day/month	233	61.8		
More than 1 day/month	46	12.2		
More than 1 day/week	78	20.7		
More than 3 days/week	20	5.3		
Working hours per week				
1-34	12	3.2		
35-39	70	18.6		
40-49	204	54.1		
More than 50	91	24.1		

### Measures

#### Basic attributes

The respondents were asked to answer questions regarding the following demographic variables: gender, age, years of service at the current workplace, years of experience, position, marital status, work shifts, frequency of exercise per week, and working hours per week.

#### Nurses' perceptions of health and productivity management

To assess hospital organizations' efforts toward maintaining nurses' health and productivity management, a questionnaire with 20 items was developed based on the indicators developed by Takahashi et al.^27)^. This served as an evaluation index of a healthy workplace culture. As our focus was on hospital organizations, the original term “company” was replaced with “hospital” or “the current workplace”; further, “employee” was replaced with “personnel.” These adjustments served to retain the original intended meanings while ensuring the questionnaire's suitability for our sample. The response method was the same as the original^27)^, with a three-point scale comprising the options of “I don't know,” “I'm not sure,” and “I know.” Similarly, a four-point scale was used to measure “Strongly disagree” to “Strongly agree.”

#### Work engagement

The Japanese version of the Utrecht Work Engagement Scale^28)^ was used. Responses were given on a seven-point scale ranging from “Not at all” to “Every day (feel it all the time).” For the analysis, the total scale scores and scores of the three sub-factors (vigor, dedication, and absorption) were used.

### Statistical analysis

First, for the question on healthy workplace culture, one group of positive answers (e.g., “I know,” “Strongly agree,” “Agree”) was designated as Recognizing Healthy Culture (RHC) and the group of negative answers (e.g., “I don't know,” “I'm not sure,” “Strongly disagree,” “Disagree”) was designated as Not Recognizing Healthy Culture (NRHC). Further, a quartile was used for WE, with the 75th percentile as the cutoff point. The group with the highest scores was designated “high work engagement” (HWE) and the group with the lowest scores was designated “low work engagement” (LWE).

Next, binomial logistic regression analysis was conducted with RHC/NRHC as the explanatory variables and HWE/LWE as the objective variable to calculate crude odds ratios (CORs) and adjusted odds ratios (AORs). For AORs, propensity scores were calculated for gender, age, years of service at the current workplace, years of experience, position, marital status, work shifts, frequency of exercise per week, and working hours per week and used as moderator variables. SPSS version 28 for Windows (IBM Corp., Armonk, NY, USA) was used for statistical analysis.

### Ethical considerations

This study was approved by the ethics committee at the Graduate School of Sports and Health Science, Juntendo University (Application Number: 2021-115). The start screen of the survey clearly stated its purpose and informed respondents that clicking the answer button implied consent to participate.

## Results

### Logistic regression analysis of nurses' perceptions of health and productivity management and work engagement

The results of the analysis are presented in Tables 2, 3-1 and 3-2. First, in the analysis using total WE scores as the objective variable, the CORs for WE tended to be higher in the RHC group than in the NRHC group for all indicator items related to healthy workplace climate. Similarly, the AORs for WE tended to be higher in the RHC group than in the NRHC group for all items except “Participation of personnel from the planning stage of the efforts” and “Reflection of personnel's opinions in the planning of the efforts.” Items with particularly high AORs were as follows: “Hospital's interest in its personnel's health” (AOR=3.25, 95% confidence interval (CI) [1.97, 5.37]); “Supervisors' support for participating in health promotion efforts during work hours” (AOR=2.69, 95% CI [1.60, 4.49]); “Active participation of the management team in these efforts” (AOR=2.68, 95% CI [1.61, 4.45]); “Colleagues' interest in health” (AOR=2.59, 95% CI [1.54, 4.33]); and “Colleagues' support for taking sick leave” (AOR=2.53, 95% CI [1.30, 4.94]).

**Table 2:** Participants' work engagement scores

	Total (n=377)				HWE (n=279)				LWE (n=98)			
	Mean	SD	Min	Max	Mean	SD	Min	Max	Mean	SD	Min	Max
Total	20.8	13.7	0	54	38.8	7.4	29	54	14.5	8.9	0	28
Vigor	6.3	4.8	0	18	11.9	2.8	9	18	3.7	2.9	0	8
Dedication	7.9	4.9	0	18	14.4	2.0	12	18	5.7	3.4	0	11
Absorption	6.6	4.7	0	18	12.9	2.5	10	18	4.3	3.0	0	9

Notes: HWE; High work engagement. LWE; Low work engagement. Cut-off point; Total = 29.0, Vigor = 9.0, Dedication = 12.0 Absorption = 10.0

**Table 3-1:** Association between health and productivity management and work engagement (logistic regression analysis)

＃	Index items for healthy workplace culture		Total							Vigor					
		n*	Crude OR	95% CI		Adjusted OR	95% CI			Crude OR	95% CI		Adjusted OR	95% CI	
1	Hospital policy on maintaining and promoting health	46	3.08	1.63	5.80	2.26	1.12	4.55		2.71	1.44	5.07	1.65	0.82	3.32
2	Procedures for handling health issues	120	2.45	1.52	3.96	1.83	1.09	3.06		3.01	1.89	4.76	2.20	1.35	3.60
3	Program and support for returning to work after a long absence	167	2.40	1.49	3.84	2.12	1.31	3.43		2.37	1.52	3.70	2.04	1.28	3.23
4	Programs and menus for improving health	77	2.18	1.27	3.71	2.00	1.15	3.48		2.46	1.47	4.12	2.19	1.28	3.73
5	Program and support for improving mental health	194	2.29	1.41	3.71	2.07	1.26	3.40		2.74	1.73	4.33	2.41	1.50	3.86
6	Feedback on physical condition from supervisors to personnel	193	2.79	1.71	4.56	2.50	1.51	4.13		3.28	2.06	5.23	2.91	1.81	4.69
7	Provision of useful information about health promotion	108	2.91	1.78	4.74	2.50	1.51	4.13		3.32	2.07	5.32	2.77	1.70	4.51
8	Communication about the importance of promoting a healthy workplace from supervisors to personnel	117	2.75	1.70	4.45	2.36	1.44	3.88		3.39	2.13	5.39	2.93	1.81	4.72
9	Participation of personnel from the planning stage of the efforts	83	1.88	1.11	3.18	1.58	0.90	2.77		3.29	1.98	5.46	2.66	1.56	4.53
10	Reflection of personnel's opinions in the planning of the efforts	98	1.67	1.01	2.77	1.40	0.83	2.36		2.22	1.37	3.58	1.83	1.10	3.02
11	Places to consult with others about health and safety issues	72	2.90	1.69	4.97	2.25	1.27	3.97		3.32	1.95	5.65	2.48	1.41	4.33
12	Hospital's interest in its personnel's health	154	3.72	2.29	6.03	3.25	1.97	5.37		4.56	2.86	7.25	3.88	2.40	6.25
13	Hospital's interest in creating a healthy work environment	157	2.64	1.64	4.23	2.18	1.33	3.56		3.66	2.32	5.77	3.03	1.89	4.86
14	Supervisors' support for participating in health promotion efforts during work hours	193	3.17	1.92	5.22	2.69	1.60	4.49		3.10	1.95	4.93	2.54	1.57	4.11
15	Active participation of the management team in these efforts	183	3.23	1.97	5.29	2.68	1.61	4.45		3.64	2.28	5.80	2.99	1.85	4.85
16	Colleagues' support for taking sick leave	285	2.88	1.49	5.56	2.53	1.30	4.94		2.26	1.27	3.99	1.94	1.08	3.48
17	Colleagues' interest in health promotion	201	3.01	1.82	4.98	2.59	1.54	4.33		3.06	1.91	4.89	2.67	1.65	4.32
18	Conversations with colleagues about health promotion efforts	114	2.43	1.50	3.94	1.77	1.05	2.98		3.43	2.15	5.47	2.43	1.47	4.00
19	Encouraging colleagues about these efforts	117	2.44	1.51	3.95	1.79	1.07	3.00		3.03	1.91	4.82	2.22	1.36	3.63
20	Participating with colleagues to promote a healthy workplace	97	2.86	1.74	4.71	2.41	1.43	4.03		3.46	2.13	5.61	2.88	1.74	4.75

Notes: For the adjusted odds ratios, propensity scores were calculated for the moderator variables of gender, age, years of service at the current workplace, years of experience, position, marital status, work shifts, frequency of exercise per week, and working hours per week. Crude OR/Adjusted OR: Odds ratio of RHC with NRHC as the reference. 95% CI: 95% confidence interval. * Number of those in the RHC group.

**Table 3-2:** Association between health and productivity management and work engagement (logistic regression analysis) continued

＃	Index items for healthy workplace culture		Dedication							Absorption					
		n*	Crude OR	95% CI		Adjusted OR	95% CI			Crude OR	95% CI		Adjusted OR	95% CI	
1	Hospital policy on maintaining and promoting health	46	4.36	2.30	8.25	2.96	1.47	5.96		2.72	1.44	5.14	1.84	0.91	3.73
2	Procedures for handling health issues	120	3.28	2.02	5.32	2.33	1.39	3.91		2.00	1.24	3.23	1.47	0.87	2.45
3	Program and support for returning to work after a long absence	167	2.71	1.68	4.38	2.35	1.43	3.84		1.96	1.23	3.12	1.65	1.02	2.67
4	Programs and menus for improving health	77	2.26	1.32	3.86	2.11	1.21	3.67		1.99	1.16	3.39	1.71	0.98	2.99
5	Program and support for improving mental health	194	2.99	1.81	4.93	2.53	1.51	4.24		1.85	1.15	2.97	1.52	0.93	2.49
6	Feedback on physical condition from supervisors to personnel	193	2.84	1.73	4.67	2.57	1.54	4.27		2.11	1.31	3.40	1.76	1.07	2.88
7	Provision of useful information about health promotion	108	3.25	1.99	5.31	2.70	1.62	4.48		2.37	1.45	3.85	1.92	1.16	3.19
8	Communication about the importance of promoting a healthy workplace from supervisors to personnel	117	2.72	1.67	4.41	2.31	1.40	3.81		2.25	1.39	3.63	1.83	1.11	3.02
9	Participation of personnel from the planning stage of the efforts	83	1.82	1.07	3.09	1.60	0.91	2.80		2.13	1.26	3.58	1.76	1.01	3.06
10	Reflection of personnel's opinions in the planning of the efforts	98	1.99	1.20	3.28	1.65	0.98	2.79		1.64	0.99	2.71	1.35	0.80	2.29
11	Places to consult with others about health and safety issues	72	2.59	1.50	4.45	1.84	1.03	3.28		2.26	1.31	3.90	1.63	0.91	2.91
12	Hospital's interest in its personnel's health	154	3.32	2.04	5.37	2.84	1.72	4.69		3.19	1.98	5.14	2.67	1.63	4.38
13	Hospital's interest in creating a healthy work environment	157	2.98	1.84	4.81	2.39	1.45	3.94		2.04	1.28	3.25	1.56	0.95	2.55
14	Supervisors' support for participating in health promotion efforts during work hours	193	3.03	1.84	5.00	2.51	1.50	4.21		3.04	1.85	4.99	2.45	1.46	4.10
15	Active participation of the management team in these efforts	183	3.29	2.00	5.40	2.64	1.58	4.43		3.11	1.91	5.07	2.49	1.50	4.14
16	Colleagues' support for taking sick leave	285	3.54	1.75	7.17	3.11	1.52	6.35		2.37	1.27	4.42	2.01	1.06	3.80
17	Colleagues' interest in health promotion	201	2.53	1.54	4.16	2.02	1.21	3.39		2.25	1.38	3.65	1.84	1.11	3.03
18	Conversations with colleagues about health promotion efforts	114	2.12	1.30	3.45	1.38	0.81	2.36		2.11	1.30	3.41	1.44	0.85	2.44
19	Encouraging colleagues about these efforts	117	3.27	2.01	5.31	2.35	1.40	3.95		1.88	1.16	3.04	1.30	0.77	2.19
20	Participating with colleagues to promote a healthy workplace	97	2.31	1.40	3.81	1.89	1.11	3.18		2.31	1.40	3.80	1.86	1.10	3.13

Notes: For the adjusted odds ratios, propensity scores were calculated for the moderator variables of gender, age, years of service at the current workplace, years of experience, position, marital status, work shifts, frequency of exercise per week, and working hours per week. Crude OR/Adjusted OR: Odds ratio of RHC with NRHC as the reference. 95% CI: 95% confidence interval. * Number of those in the RHC group.

Second, analysis was conducted using vigor as the objective variable. The COR for vigor tended to be higher in the RHC group than in the NRHC group for all indicator items related to healthy workplace climate. The AORs of WE tended to be higher in the RHC group than in the NRHC group for all items except “Hospital policy on maintaining and promoting health.”

Third, analysis was conducted using the dedication sub-factor as the objective variable. The CORs for dedication tended to be higher in the RHC group than in the NRHC group for all indicator items related to healthy workplace climate. The AORs for WE tended to be higher in the RHC group than in the NRHC group for all items except “Participation of personnel from the planning stage of the efforts,” “Reflection of personnel's opinions in the planning of the efforts” and “Conversations with colleagues about health promotion efforts.”

Finally, analysis was conducted using the absorption sub-factor as the objective variable. The CORs for absorption tended to be higher in the RHC group than in the NRHC group for all items except “Reflection of personnel's opinions in the planning of the efforts.” The AORs for WE tended to be higher in the RHC group than in NRHC group for “Program and support for returning to work after a long absence,” “Feedback on physical condition from supervisors to personnel,” “Provision of useful information about health promotion,” “Communication about the importance of promoting a healthy workplace from supervisors to personnel,” “Participation of personnel from the planning stage of the efforts,” “Hospital's interest in its personnel's health,” “Supervisors' support for participating in health promotion efforts during work hours,” “Active participation of the management team in these efforts,” “Colleagues' support for taking sick leave,” “Colleagues' interest in health promotion,” and “Participating with colleagues to promote a healthy workplace.”

## Discussion

In this study, we examined the relationship between nurses' positive attitude toward WE and the efforts made by hospitals (employers), supervisors (e.g., nursing departments, managers, and chiefs), colleagues, and other individuals in hospital organizations to promote health and productivity management. This section examines the relationship between WE and four items that showed high overall AOR scores in our findings: “Hospital's interest in its personnel's health,” “Supervisors' support for participation in health promotion efforts during work hours,” “Active participation of the management team in these efforts,” and “Colleagues' interest in health.”

### Hospital's interest in its personnel's health

To promote new measures in health and productivity management, the need for a corporate culture that accepts new programs and a workplace environment where supervisors and colleagues can use the programs is suggested^29)^. In other words, to achieve organizational results, employees must participate in health promotion efforts. Employees' thoughts and feelings cannot be controlled directly^26)^, but can be addressed indirectly through changes in the workplace environment. In our study, the item “Hospital's interest in its personnel's health” had the highest AOR (2.67). The process of measuring WE involves the evaluation of the values employees perceive in an organization's actions against its espoused values to determine sincerity and against personal values to determine congruence^30)^. The results suggest that hospitals' concern for the health of their employees contributes effectively to the improvement of their WE. Based on these results, we believe that hospitals need to create a work environment that affirms health management, explains resource use, communicates a clear vision, and makes health goals known^31)^ throughout the organization. We believe that this will increase trust in the hospital as a workplace where employees' health is protected.

Thus, the results suggest that acceptance by the hospital organization of health and productivity management and the creation of a work environment where the use of resources is explained—as well as a clear vision and health goals are communicated^31)^—across the organization lead to a good evaluation of the hospital and contribute toward improving nursing personnel's WE.

### Supervisors' support for health promotion efforts during work hours

Studies comparing employees' participation rates in health programs have shown higher participation rates among employees of small- and medium-sized companies compared to among employees of large companies^32)^. The suggested reason for these findings is the rapid communication of knowledge and motivation because of a smaller staff in small- and medium-sized companies' networks. Nursing organizations tend to be organized into nursing units, such as hospital wards^33, 34)^, where nurses are assigned under the supervision of a nurse manager. The staffing levels (organizational design) of these organizations are similar to those of small businesses with fewer than 50 employees. Higher levels of engagement have been observed when supervisors exhibit relationship-related behaviors^35)^. The current study also showed a significant difference for the item related to supervisors' support for health promotion efforts during work hours (AOR, 2.69), suggesting that supervisors' support for such efforts increases nurses' WE. In the organizational design of nursing organizations, we believe that the actions of the supervisor—the head nurse—build the organizational climate. We infer that in an organization where the supervisor's support for health activities is demonstrated without discomfort, employees feel secure and form a sense of belonging. We believe that the formation of such a sense of belonging will enhance WE.

### Active participation of management team in health promotion efforts

A good work environment is essential for workplace health promotion efforts to be effective^36)^, and the outcomes of such health promotion programs depend on organizational factors such as the top management's leadership^24)^. Berry et al.^37)^, in their discussion on “engaged leadership at multiple levels,” suggested that if chief executive officers find time for exercise, employees would be less reluctant to take a fitness leave. In other words, creating a work environment that allows people to participate willingly in organizational efforts, explaining the use of resources, communicating a clear vision and health goals, and demonstrating exemplary behavioral changes by the top management will likely lead to successful health and productivity management^38)^. In this study, the item related to the active participation of the top management team in the health promotion efforts of the organization showed significant results regarding WE (AOR, 2.68), followed by the AOR of the item on supervisors' support for these efforts. As an intervention process to improve WE, Bakker and Leiter^30)^ reported that, “In the initial phases of a new initiative to enhance WE, this role could be effectively served by the CEO,” which is consistent with our results. Thus, it is suggested that WE is associated with specific behaviors (e.g., expression of concern about personnel's health) by physicians in management and leadership roles, heads of nursing organizations, and nurse directors. Creating a safe environment for engaging in health activities will lead to a positive attitude toward the workplace. We believe that this sense of being protected by the workplace will increase nurses' WE.

### Colleagues' interest in health promotion

Interactions with social networks of friends and colleagues^38, 39)^ and communicating about health^40)^ are believed to contribute to behavioral changes. Thus, a growing interest in health among colleagues seems to create a positive work environment, including helping promote support for each other's physical condition. In our findings, colleagues' interest in health was significantly associated with WE (AOR, 2.59), corroborating the findings of the cited research and indicating an association between colleagues' interest in health and individual WE. In another study, an increase in WE due to individual health efforts seemed to influence nurses^41)^ and lead to the creation of a vibrant and rewarding work environment. The nature of nurses' work tends to hinder their ability to focus on their own health during working hours. However, crossover effects^42-45)^ can be expected by building a support system that provides opportunities to discuss club activities in hospitals and encourages efforts to improve an individual's health. Thus, although it can be extremely difficult^46)^ to change established lifestyles and exercise habits, if energetic colleagues can inspire other nurses and help the latter imagine themselves as energetic, this process can improve the awareness and importance of physical exercise and health, and eventually of WE.

### The effects of improving WE in health management

Regarding the effects of health management initiatives, organizations with higher rates of employee participation have a positive impact on presenteeism^32)^. Furthermore, presenteeism is negatively correlated with WE and satisfaction^47)^.

We believe that if presenteeism can be reduced through health management, it may positively change the attitude toward work and contribute to an improvement in WE. Active participation of management was significant in the hospital organization that we studied. Previous studies that have examined the relationship between presenteeism, climate, and organizational support for nurses have also linked organizational support to the risk of presenteeism^42)^. Until now, the retention and prevention of turnover of nurses has required the development of a work environment that allows nurses to continue working, including improvements in working hour management, night shift systems, and occupational health and safety^48)^. However, the Ministry of Health, Labour and Welfare's recent survey of reasons for leaving the workforce^49)^ shows that the top reason for turnover is physical and mental health. These findings suggest that efforts to engage in health activities through health management may lead to improved effects on physical and mental health and WE, and may contribute to the prevention of nurse turnover and retention.

## Conclusion

Reducing the high nurse turnover rate in Japan is an important concern. The impact of the nursing shortage, due to a declining workforce and falling birth rate, on patient care is multifaceted^50)^. We argue that health and productivity management initiatives in hospital organizations may have a positive impact on nurse WE. Other major implications of the survey results are that the enhancement of health promotion support systems^51)^ and the development of a highly energetic and enthusiastic work environment in the field may contribute to promoting a rewarding workplace, improving retention rates, and preventing turnover.

## Limitations

As this study is based on an online questionnaire survey, it does not reveal the specific health activity initiatives required for health management. We believe that future qualitative surveys, such as interviews, will reveal a more detailed relationship between the perception of health management initiatives and WE. Analyzing the relationship between specific initiatives and WE will lead to expectations for further development of future research on health management. Furthermore, we believe that the activation of health activities in hospital organizations, not only among nurses, will enhance WE among all healthcare workers, leading to their retention in the workplace and prevention of their intention to leave the workforce. We believe that this will lead to new insights into future work styles in the medical field.

## Scope for further research

Although the number of medical corporations certified for health management is increasing, a measurement scale for health activity initiatives in hospital organizations has not yet been developed. Therefore, we employed health culture indicators in corporate organizations to measure perceived health efforts. In the future, it is necessary to develop a scale to measure health activity efforts in highly specialized organizations such as medical corporations and social medical corporations. In addition, this study revealed that the relationship between awareness (perception) of health activity efforts and WE is highly significant. In the future, it will be necessary to further explore the factors necessary to continue working by focusing on the relationship between vitality, enthusiasm, and immersion, which are sub-factors of WE.

## Funding

The authors received no financial support for the research.

## Author contributions

IK performed the data analysis; IT, HY, MY, and MM interpreted the analysis results and contributed significantly to the writing of the manuscript. All authors read and approved the final manuscript.

## Conflicts of interest statement

The authors declare that they have no conflicts of interest.
